# Modifiable unhealthy lifestyle behaviours in subclinical manifestations of attention-deficit hyperactivity disorder: what are the first empirical results and putative clinical implications?

**DOI:** 10.1192/bjo.2024.837

**Published:** 2025-03-20

**Authors:** Kristin Annawald, Thomas Meyer

**Affiliations:** Department of Psychosomatic Medicine and Psychotherapy, University Medical Centre Göttingen, University of Göttingen, Germany

**Keywords:** Attention-deficit hyperactivity disorder (ADHD), unhealthy lifestyle behaviours, eating disorders, media use, subclinical manifestations

## Abstract

Attention-deficit hyperactivity disorder (ADHD) is one of the most common mental disorders in adolescents, and a full syndrome diagnosis requires a combination of persistent symptoms. In a multicentre cross-sectional study from Italy using a non-clinical sample from a secondary school comprising 440 adolescents, published in this issue of BJPsych Open, Gostoli et al examined whether unhealthy lifestyle habits are linked to both clinical manifestation of ADHD and subclinical symptomatology. In line with the literature, the authors demonstrate an association between clinical ADHD diagnosis, unhealthy lifestyle behaviours and psychosocial impairments. Modifiable, adverse lifestyle behaviours are also prevalent in subclinical ADHD manifestations. This observation may be important for child and adolescent psychiatry when considering targeted health promotion approaches that delay or prevent progression from subclinical to clinical ADHD. In this article, we discuss from a clinical perspective the putative relevance of addressing subclinical ADHD symptoms in the context of the existing literature.


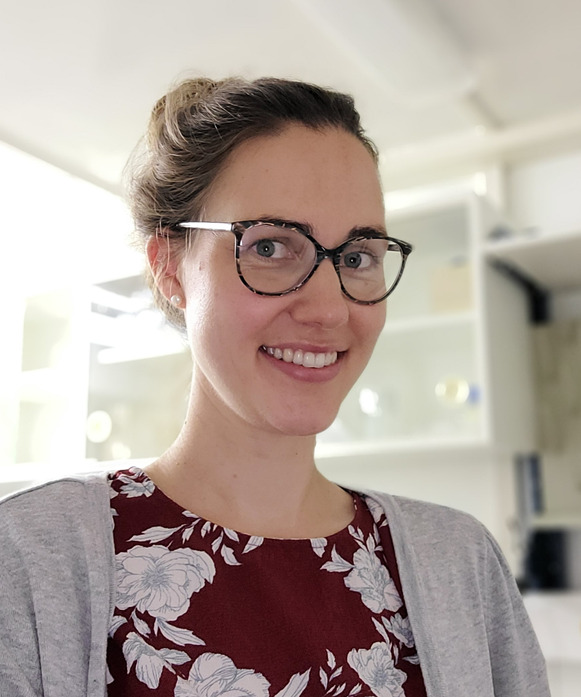


We read with interest the important work by Gostoli and colleagues demonstrating an association between attention-deficit hyperactivity disorder (ADHD) and unhealthy lifestyle behaviours in a sample of 440 Italian adolescents with a mean age of 14 years.^1^ Within this study cohort of upper secondary school students, the authors found that, similar to clinical ADHD, subclinical manifestations were also associated with several unhealthy lifestyle behaviours, including altered eating patterns, problematic smartphone use and impaired sleep quality. Subclinical ADHD refers to subthreshold symptom expressions with distinct ADHD features which are not severe, numerous or impairing enough to warrant a DSM-5 diagnosis. Nevertheless, subclinical ADHD may have a significant impact on various aspects of daily life.

The authors showed that participants classified as the clinical ADHD group had a significantly higher frequency of binge drinking than both the subclinical group and the group with no ADHD. Total scores from the Internet Addiction Test as well as scores from the Compromised Social Quality of Life instrument were significantly different (*P* < 0.001) between the three groups based on ADHD symptom severity, with the group of manifest ADHD reporting greater impairments. In addition, daily hours of sleep and quality of sleep measured with an item from the Pittsburgh Sleep Quality Index differed significantly (*P* < 0.001) between the groups, with the group with clinically manifest ADHD sleeping fewer hours and having lower scores. By using a clinimetric assessment to identify a variety of lifestyle factors, this study expands our knowledge of a range of modifiable individual risk behaviours in adolescents with ADHD symptoms, even though the threshold for a full syndrome diagnosis on an individual basis was not met.^1^ As outlined by the authors, the study by Gostoli et al is limited by its observational study design, which does not allow for causal conclusions between subclinical or manifest ADHD and unhealthy lifestyle behaviours. Further limitations arise from the fact that ADHD was not clinically diagnosed by mental health professionals and that only self-assessments were used for group assignment and lifestyle behaviour classification. The study is limited by its exclusively descriptive statistical analysis, which does not allow adjustment for clinically relevant confounders such as gender in a multivariate approach.

Using the dichotomised DSM-5 diagnostic criteria for ADHD, numerous studies have compared the diagnosed ADHD group with healthy control individuals, with most of these studies ignoring the undetermined number of subclinical cases. Using these dichotomised diagnostic criteria, Bourchtein et al reported that, while technology use is more common in adolescents with ADHD, it is also associated with more sleep problems and reduced sleep duration in the night, regardless of ADHD status.^2^ It is known that individuals with ADHD often report daytime sleepiness and have circadian rhythm abnormalities and other sleep disorders.^3^ Using principal component analysis to characterise dietary patterns, Rojo-Marticella et al found an association between ADHD diagnosis and dietary habits measured by a food consumption frequency questionnaire in two samples of paediatric probands of different ages.^4^ However, the authors also found that children with inattentive presentation, which may represent subclinical manifestations, had a higher risk of unhealthy eating habits (high-sugar, high-fat diets and lower nutritional quality) than the control group. In a nationally representative sample of young adults, Bleck and co-authors observed that people with subclinical ADHD status were more likely to have a subclinical bingeing and/or purging eating disorder than control individuals without ADHD symptomatology, but did not differ with respect to subclinical restrictive eating disorder, as assessed by a self-reported questionnaire for eating disorders.^5^ In addition, children and adolescents with ADHD symptoms appear to be more prone to intensive use of digital media, including smartphones and the internet.^6^

Compared with diagnosed people with ADHD, individuals with subsyndromal ADHD symptoms generally receive less attention from the medical profession and society, although the prevalence rate of subthreshold ADHD may be higher compared with people with the full syndrome.^7^ Individuals with subclinical ADHD symptoms may less frequently seek medical care and do not receive enough medical attention compared with people with a full symptomatology, resulting in longer waiting times and lower attendance at medical appointments.^8^ In individuals with a milder symptom severity, the development of the manifest disorder may take longer, suggesting that the progression to a complete symptomatic manifestation may be delayed or even impeded in childhood, adolescence or early adulthood. Whereas some individuals never reach full symptom expression to merit a diagnosis, others may oscillate between subsyndromal and full symptomatology over the course of adolescence and adulthood. Although subclinical ADHD and clinically diagnosed ADHD have similar core symptoms, their severity and clinical expression can differ considerably. It is also difficult to distinguish the healthy from the subclinical group.

Regarding the development of subclinical and clinical ADHD, research on the effects of addictive eating behaviour, digital screen use, physical activity and sleep deprivation on adolescent health is still in its infancy, as the neurodevelopmental consequences of these stressors are not yet understood.^2,4^ Numerous studies have shown that ADHD symptoms in both children and adolescents are associated with maladaptive stress coping strategies and impairment in daily life in various domains.^9^ Unhealthy lifestyle habits including inappropriate media consumption, limited sports activity and altered sleep habits may contribute to the development of inattention and/or hyperactivity symptoms in children and adolescents, leading to additional long-term health issues affecting scholastic outcomes.^10^

Previous literature has studied unhealthy lifestyle habits in people with ADHD, including data from the population-based cross-sectional KiGGS (German Health Interview and Examination Survey for Children and Adolescents) study. This nationwide survey demonstrated that, in participants aged 6 to 17 years, parent-rated scores on the hyperactivity/inattention subscale of the Strengths and Difficulties Questionnaire (SDQ-HI) used as a continuous measure of ADHD symptoms were associated with prolonged television consumption and poor diet quality. The authors of this KiGGS substudy, van Egmond-Fröhlich et al, also reported that the significant positive association between SDQ-HI scores and energy intake from beverages was due to both increased volume and high calorie content.^11^

Clinical ADHD is also related to eating disorders such as bulimia nervosa and binge eating which both indicate a loss of control over excessive food intake and ultimately lead to obesity.^12^ Impulsivity, a hallmark symptom of manifest and subclinical ADHD, increases the risk of excessive alcohol consumption and makes adolescents diagnosed with ADHD more susceptible to alcohol use disorders.^13^ The occurrence of food cravings and high impulsivity are associated with changes in the reward system, as awareness of and attention to overeating are suppressed and, as a result, large amounts of food or alcohol are consumed in a short period of time.^14^ Addictive eating habits and other unfavourable lifestyle habits are overrepresented in individuals diagnosed with ADHD, probably because of a lack of control of food intake through disinhibition resulting from alterations in mesolimbic dopamine signalling.^14^

The research in this study benefits from the concept of subclinical disorders, which was introduced in the early 1990s to overcome the limitations of a categorical approach to diagnosing specific psychiatric illness entities in individuals who do not meet full diagnostic criteria according to the prevailing classification system.^7^ Whereas a categorical approach to assessment relies on diagnostic criteria that determine the presence or absence of a predefined pattern of specific symptoms, a dimensional approach assesses symptom severity on a continuous non-dichotomised spectrum. Clinical studies of psychiatric disorders such as ADHD, which have a wide overlap with subthreshold manifestations, may benefit from the use of non-dichotomised measures to overcome the limitations of a binary classification system.^15^ Classification as subthreshold ADHD is based on the presence of some inattentive, impulsive and/or hyperactive symptoms that in sum do not fulfil all the requirements for a clinical diagnosis. The frequency of diminished cognitive control and attention functioning in population-based samples suggestive of subclinical ADHD has not yet been established, and a subset of children and adolescents with intellectual disability, internalised and externalised behaviour problems, and psychosocial maladjustment may fall into the category of subclinical ADHD.

As the study by Gostoli and colleagues shows, a higher prevalence of unhealthy lifestyle behaviour is also observed in individuals with subclinical manifestations of ADHD symptoms.^1^ The clinical significance of this observation cannot be overstated, as an unknown percentage of children and adolescents with uncontrolled eating behaviour, sleep problems and/or unlimited smartphone use may be overlooked as subclinical ADHD cases, on the assumption that they are solely suffering from the negative side effects of their unhealthy lifestyle habits. Mental health professionals should consider unhealthy lifestyle behaviour as a potential early sign of subclinical or clinical ADHD. This could lead to mental health professionals becoming more sensitive to identifying individuals at risk who may develop a manifest disorder in the near future. However, particular caution is required in prevention strategies not to over-diagnose the disorder or stigmatise individuals, as the causes for unhealthy lifestyle behaviour can be manifold. Future research is needed to establish whether unhealthy lifestyle habits are risk factors and/or early signs of the disorder.

A comprehensive understanding of the pathophysiological mechanisms underlying such unhealthy behaviour could help refine diagnostic criteria and promote individualised prevention and treatment strategies. Recommendations to change unfavourable lifestyle habits may ultimately improve the health-related quality of life in this group of adolescents at risk of developing the fully fledged ADHD syndrome.

## Data Availability

Data availability is not applicable to this article as no new data were created or analysed in this editorial.
